# IoT-based system of prevention and control for crop diseases and insect pests

**DOI:** 10.3389/fpls.2024.1323074

**Published:** 2024-02-01

**Authors:** Zhibin Wang, Xiaojun Qiao, Ying Wang, Hao Yu, Cuixia Mu

**Affiliations:** ^1^ Information Technology Research Center, Beijing Academy of Agriculture and Forestry Sciences, Beijing, China; ^2^ Beijing Engineering Research Center of Agricultural Internet of Things, Beijing, China; ^3^ College of Data Science and Information Technology, China Women’s University, Beijing, China

**Keywords:** ozone, Internet of Things (IoT), diseases, insect pests, pesticide-free, light trap, remote control

## Abstract

Environmentally friendly technologies for the prevention and control of crop diseases and insect pests are important to reduce the use of chemical pesticides, improve the quality of agricultural products, protect the environment, and promote sustainable development of crop production. On the basis of Internet of Things (IoT) technology, we developed a prevention and control system for crop diseases and insect pests with two main components: a plant protection device (the hardware) and an information management system (the software). To be suitable for both facility- and field-based production scenarios, we incorporated two types of plant protection devices, utilizing ozone sterilization and light-trap technologies. The devices were equipped with various sensors to realize real-time collection and monitoring of data on the crop production environment. The information management system has an IoT-based architecture and includes a mobile device app to enable remote control of the plant protection devices for intelligent management of plant protection data. The system can achieve efficient management of large-scale equipment applications and multi-device collaborative work to prevent and control pests and diseases. The developed system has operated successfully for several years in China and has been applied to cucumber, tomato, rice, and other crops. We demonstrate the effectiveness and practicality of the system in a greenhouse facility and in the field.

## 1 Introduction

Crop diseases and insect pests, which are characterized by their high diversity, serious impacts, and localized outbreaks, can cause severe agricultural losses. Diseases and pests are important factors that threaten food security, and restrict the quality and yield of crops (Carvajal-Yepes et al., 2019; Singh et al., 2021; Zheng and Zhang, 2022). Globally, 20%–40% of crop plant yields are lost annually because of the incidence of crop pests and diseases (Karmakar et al., 2022). In China, insect pests and diseases cause the annual loss of approximately 40 million tons of grains (Gao et al., 2020). Changes in the global climate and farming systems have led to changes in the incidence of agricultural diseases and insect pests. The impacts on agricultural production are increasing in severity and are even extending to previously unaffected regions (Hirschi et al., 2012; Deutsch et al., 2018). Pesticides are commonly used to control diseases and insect pests in agriculture because they are highly and rapidly effective and allow agricultural producers to increase crop yields. However, excessive application of chemical pesticides can lead to problems, such as environmental pollution, pesticide residues, and bacterial drug resistance, that seriously affect the quality and safety of crop products, the ecological environment, and sustainable agricultural development (Schreinemachers et al., 2020; Song et al., 2020; Katsoulas et al., 2021; Mack et al., 2023). Therefore, alternatives to pesticide use in agriculture are urgently required. However, scientific research and practices in plant protection and development at present are mainly conducted from the perspective of biological sciences, e.g., biochemistry, biology, and ecology. Environmentally friendly technologies also include those that use physical means, such as light, heat, electricity, mild radiation, and mechanical equipment, to prevent and control diseases and insect pests. Physical control technologies reduce the amounts of chemical pesticides required to achieve high yields in agricultural production (Vincent et al., 2009; Sang et al., 2022).

Ozone (O_3_) is a strong oxidizing agent that can kill many types of causal organisms. Moreover, ozone is rapidly decomposed to oxygen and is non-polluting (Isikber and Athanassiou, 2015; Abdelfattah et al., 2021; Wang et al., 2022a). Ozone generation by electrical discharge in air (corona-discharge) is the most commonly used method of production and has several advantages over alternative methods, including greater sustainability of the unit, higher ozone production, and higher cost-effectiveness. Because ozone can be generated easily at treatment sites using only electricity and air, it has been used extensively in the food industry (Pandiselvam et al., 2019; Pandiselvam et al., 2020) for air disinfection in closed spaces (Alimohammadi and Naderi, 2021; Epelle et al., 2022), as well as for wastewater treatment (Foroughi et al., 2022; Morrison et al., 2022).

The use of ozone gas for infection prevention and control in crop production has become an important research focus. Szumigaj-Tarnowska et al. (2020) evaluated the fungicidal effects of ozone on fungal pathogens of common mushrooms. Their experimental results demonstrated the usefulness of ozone as a disinfectant for empty growing rooms after the completion of a mushroom cultivation cycle. Fujiwara and Fujii (2002) investigated the effects of spraying ozonated water on cucumber leaves that were severely infected by powdery mildew and showed that ozonated water was at least a partially effective alternative to agricultural chemical fungicides. Zhang et al. (2021) demonstrated that using ozone water for disinfection of greenhouse soil was effective against soil microorganisms and nematodes, without producing harmful by-products. Fernández et al. (2019) applied ozone treatment to inactivate *Meloidogyne enterolobii* eggs. These authors showed that ozonation was an effective and promising method for nematode control in irrigation water, and that it reduced tomato crop infestation caused by plant-parasitic nematodes. Díaz-López et al. (2022) assessed the impact of irrigation with ozonated water on the microbial community in a Mediterranean soil, and on tomato agrophysiology and productivity in a greenhouse experiment. The authors reported that the biomasses of gram-negative bacteria and fungi were decreased by intermittent and continuous irrigation with ozonated water, and that the diversity, structure, and composition of the soil microbial community were not affected by the ozone treatments. Furthermore, soil health and fertility were not compromised. For improved application of ozone plant protection technology, specialized agricultural ozone sterilization equipment has been developed. Ebihara et al. (2013) developed a portable ozone-mist sterilization system to exterminate harmful insects in agricultural fields and greenhouses. The authors showed that aphids were exterminated in 30 s with no noticeable damage to the plant leaves. Takayama et al. (2006) developed an ozone generation system for soil sterilization and demonstrated that ozone treatment of agricultural soil reduced the abundance of soil bacteria and *Fusarium oxysporum*. Qiao et al. (2020) designed an ozone sterilization device for treatment of an organic matrix and achieved sterilization frequencies for bacteria and fungi of 88.9% and 97.9%, respectively, thus meeting the production needs.

The aforementioned studies show that ozone and ozonated water are effective in the control of fungi, bacteria, viruses, protozoa, and insect pests; however, the concentrations of ozone used, the modes of ozone production, and the methods of ozone application vary greatly among these studies. There remains a lack of ozone plant protection technologies and equipment that are easy to operate, allow for precise use, and are remotely controlled, which limits the large-scale application of ozone in agricultural plant protection.

A light trap is a physical device that is widely used for insect pest control in agriculture (Liu et al., 2013; Nielsen et al., 2013; Zhou et al., 2018; Erler and Bayram, 2021). Light traps attract and confine nocturnal insects by positive phototropism. Various types of insecticidal lamps are commonly used in crop production, including black light lamps, vibration-type insecticidal lamps, light-emitting diode (LED) insecticidal lamps, and solar insecticidal lamps. Li et al. (2019) reviewed the characteristics and applications of state-of-the-art insecticidal lamps in agriculture in China. Tu et al. (2016) developed an LED multispectral circulating solar insecticidal lamp that can control the time cycle and open a different spectral peak lamp to kill insects at night. The authors showed that the lamp effectively killed pests of rice, thereby reducing the amount of pesticides used in rice production and improving economic efficiency. On the basis of interspecific differences among insects in the phototactic response to diverse light of narrow wavelength, Bian et al. (2018) designed a light source friendly to natural enemies of pests using LED chips. Carvalho et al. (2021) used a smart LED lamp to monitor insect populations. Han et al. (2020) conducted a field experiment to investigate the control range and effectiveness of a suction solar energy insecticidal lamp on Asian corn borer *Ostrinia furnacalis*. The authors observed that the lamp trapped insects in 40 families in nine orders; the effective control distance to *O. furnacalis* was approximately 60 m, and the control area of a single lamp was about 1.1 hm^2^. Lam et al. (2013) used light traps to monitor and control brown planthoppers in the Mekong Delta; a light trap may destroy millions of brown planthoppers per night during peak population abundance. Wang et al. (2023) established a green and efficient light trapping and control technology for insect pests in peanut fields. The effects of different light sources and the weather on the trapping efficiency of solar light traps were analyzed. Their results showed that solar insect light traps were effective in trapping peanut insect pests with minimal negative impact on natural enemy insects and neutral insects, and thus can be used as a green control method against peanut insect pests.

To the best of our knowledge, insecticidal lamps in current use are predominantly operated in unattended fields in remote geographical locations, making fault detection and maintenance difficult. Furthermore, most insecticidal lamps in China are independently installed and manually debugged. No unified and shared information management system has been established, and therefore users are unable to monitor the real-time working status of the insecticidal lamps, which reduces their usage efficiency. In addition, solar insecticidal lamps are mainly used for the prevention and control of pests in fields, whereas their application in pest control within facilities is relatively limited. In particular, insecticidal lamps are generally ineffective for disease prevention and control.

The Internet of Things (IoT) is a network of devices or things that communicate with each other and with the surrounding environment and share information through the Internet (Tzounis et al., 2017; Khanna and Kaur, 2020; Kagan et al., 2022). IoT devices are programmed to perform tasks to minimize human effort. In particular, IoT can provide solutions for many problems in the absence of human attendance. When used in agriculture, IoT improves the quality of farming outcomes (Khattab et al., 2019; Terence and Purushothaman, 2020; Gaikwad et al., 2021; Tashakkori et al., 2021; Sayem et al., 2023).

The objectives of the present study were to develop a green prevention and control technology for crop diseases and insect pests to reduce the amounts of chemical pesticides used and to promote sustainable crop production. Thus, in the present study, we combined ozone sterilization, light trap, and IoT technologies, and developed a system for the prevention and control of crop diseases and insect pests to overcome the aforementioned problems. The main contributions of this work are as follows.

1) Two types of plant protection devices were designed: one suitable for production facilities and one for field use. Ozone sterilization and light-trap technologies were used to prevent and control crop diseases and insect pests, which reduced the need to apply chemical pesticides during crop production.
2) Light intensity, temperature, and relative humidity sensors, and a surveillance camera were incorporated with the plant protection devices to realize real-time collection and monitoring of environmental data. The information generated can be used by farmers to determine the growth status of crops and the occurrence of diseases and pests in real time and adjust the working mode of the plant protection devices to achieve rapid and accurate prevention and control of crop diseases and pests.
3) An IoT-based information management system was developed for remote control of the plant protection devices and the intelligent management of plant protection data. This system can be applied to unify the scheduling of plant protection operations and multi-device collaborative work, thereby improving the usage efficiency of the plant protection equipment and reducing human resources.

The remainder of this paper is organized as follows. The system’s architecture, hardware, and software are described in Section 2. Section 3 presents the experimental results. A discussion and future work are given in Section 4. Finally, the conclusions are provided in Section 5.

## 2 Materials and methods

### 2.1 System overview

**Figure 1:**
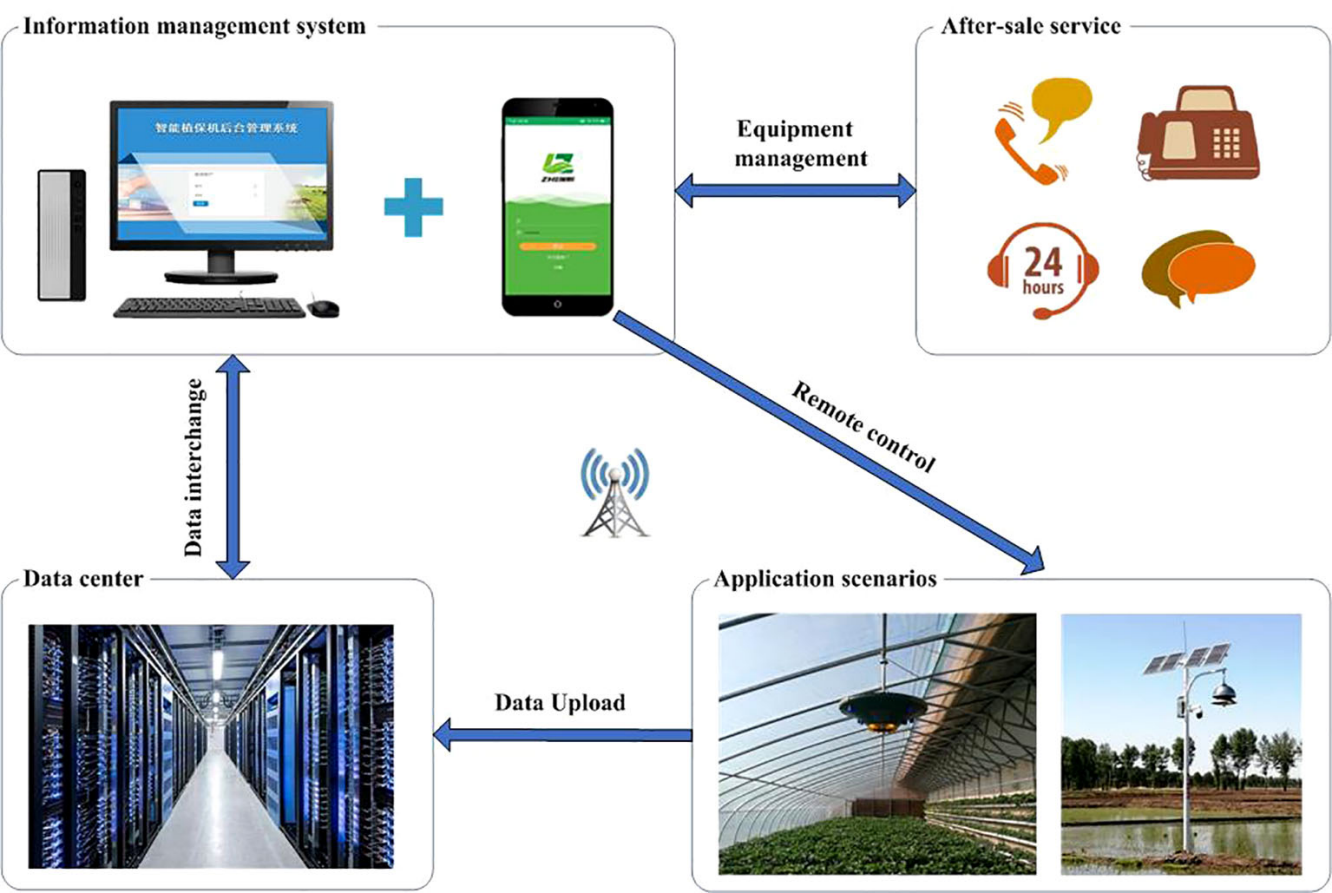
Overview of the IoT-based prevention and control system for crop diseases and insect pests.

An overview of the IoT-based prevention and control system for crop diseases and insect pests is provided in **Figure 1**. The system comprises four main modules: plant protection device, information management system, data center, and after-sale service.

Two types of plant protection devices were designed to achieve the prevention and control of crop diseases and insect pests in different production scenarios, namely, a facility plant protection device and a field insecticidal plant protection device. The plant protection devices communicate with the information management system through a wireless transmission network. Users can operate the device remotely using a mobile app for real-time adjustment of the fan wind speed and ozone release, collection of environmental data, opening of the trap lamp, and other functions. The information management system has an IoT-based architecture, and includes a web-based information management system and a mobile app. The web-based information management system interacts mainly with the data center for intelligent management of plant protection data. The mobile app is used mainly for remote control, data query, and equipment maintenance for intelligent management of hardware devices. The main functions of the after-sale service module are to provide technical support in relation to the plant protection devices, including introduction, delivery, installation, commissioning, maintenance, technical training, and on-site service. The technical support assists farmers in using the devices accurately in actual agricultural production. All plant protection data, including environmental data, equipment information, and plant protection operation records, are stored in the data center.

In practical application, the detailed procedure of the system’s operation is as follows. (1) After the device is installed and powered on, the operator uses a mobile phone to scan the QR code on the device, then download and install the mobile app for use on the device. (2) The user registers his device through a usage account, and enters and sets the usage information according to the prompts from the mobile app, such as basic information on the plant protection device, crop information, application scenarios, device operation modes, and the user’s personal information. (3) After providing the device usage information, the device can be launched and run. Simultaneously, the plant protection device establishes a TCP connection with the backend gateway of the information management system through the wireless network for real-time transmission of plant protection data and operation instructions. The web-based information management system interacts with the data center to manage the received data, such as data queries, export, and visualization. (4) During the running of the device, users can check the working status and adjust the working mode of the device at any time through the mobile app. If the device malfunctions, users can enter the device’s fault information online. Staff can promptly maintain or replace the faulty equipment, ensuring that it does not affect crop production. (5) When multiple plant protection devices are installed and used in different areas and application scenarios, the user can manage all devices under their account in real time and remotely control multiple devices through the mobile app for collaborative pest and disease prevention and control. For example, when multiple plant protection devices are installed on a property, the user can adjust the working modes of all devices in batches or separately according to application needs, which enables combination of all devices operating together or several devices operating alternately, thereby achieving remote scheduling and collaborative operation of multiple devices, and jointly achieving disease and pest prevention and control.

### 2.2 System architecture

**Figure 2:**
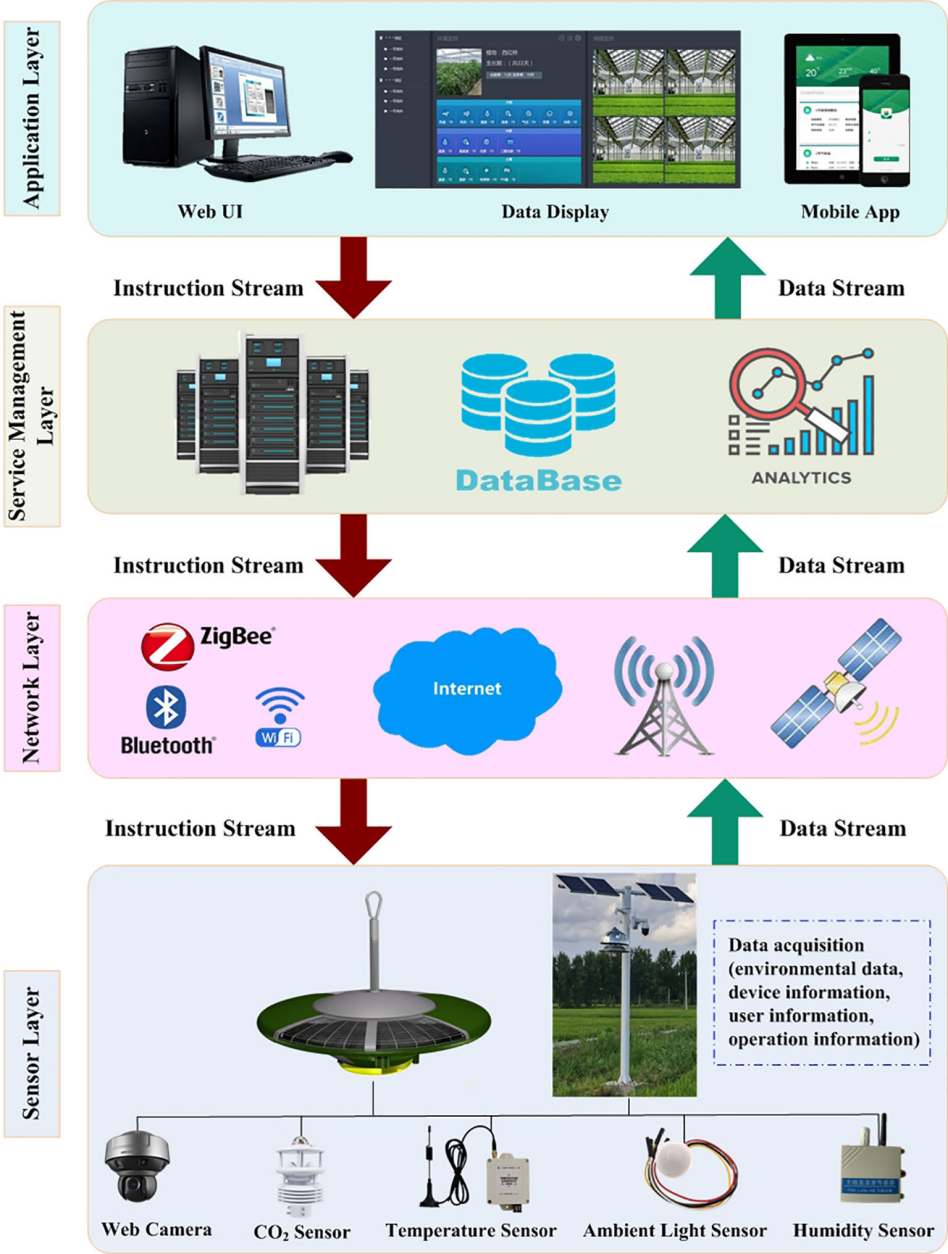
Architecture of the IoT-based prevention and control system for crop diseases and insect pests.

The system architecture has four main layers, namely, sensor, network, service management, and application layers (**Figure 2**).

#### 2.2.1 Sensor layer

The sensor layer consists of sensors that collect information from the surroundings and actuators that transmit the information through a gateway and network for further processing. Different kinds of sensors can be deployed in the plant protection device, including light intensity, temperature, relative humidity, and CO_2_ sensors, and surveillance cameras. The specifications of these environmental sensors are summarized in **Table 1**.

**Table 1:** Environmental sensor specifications.

Sensor	Model	Monitoring principle	Range	Accuracy	Communication protocol
Air temperature	SHT30	Heat conduction	-40 to 125°C	± 0.3	I^2^C
Air humidity	SHT30	Capacitance measurement	0-100%RH	± 2%	I^2^C
CO_2_ concentration	MG811	Solid electrolyte cells	0-10000ppm	± 50	RS485
Light intensity	BH1750FVI	Photovoltaic effect	1-65535Lx	0.96-1.44	I^2^C
Web camera	DS-2DE4423IW-D	Converting optical signals into electrical signals for storage	< 150m	4 million pixels	802.1X

#### 2.2.2 Network layer

The network layer is the coordination layer between the sensor layer and the service management layer, and is responsible for data transmission tasks. The network layer has data access and data transmission functions, and transfers data created by the sensor layer through secure channels to the service management layer. Wireless technologies, such as ZigBee, RFID (radio frequency identification), Wi-Fi, and GPRS (General Packet Radio Services), are used to transmit data. Considering factors such as the crop production environment and cost, the network layer used in the system is a close-range wireless network combined with remote wireless communication. The transferred information is mainly the collected environmental data, device information, personnel information, operation and maintenance information, and device control instructions. The procedure by which the plant protection devices communicate wirelessly with the information management system is as follows. (1) Establishing a TCP connection: after the plant protection device is powered on and running, it connects to the Internet through a wireless network, and attempts to establish a TCP connection with the backend gateway of the information management system. TCP connections are used to ensure the reliability and orderliness of data transmission. (2) Handshaking procedure: the plant protection device sends a TCP SYN packet to the backend gateway of the system, requesting the establishment of a connection. After receiving the SYN packet, the backend gateway responds with a SYN-ACK packet to confirm the connection request. The handshake process is complete. (3) Transmission, parsing, and processing of private protocol data: after establishing a TCP connection, the plant protection device sends private protocol data to the backend gateway of the system. The backend gateway receiving the private protocol data needs to parse the data, then performs data verification based on the parsed data and instructions, and executes corresponding operations, or generates control instructions to send back to the plant protection device. (4) Data encryption and secure transmission: to ensure the security of the data, the private protocol data should be encrypted during transmission. Common encryption algorithms include AES and RSA. In addition, security protocols, such as SSL/TLS, can be used to protect the data integrity and confidentiality. (5) Close TCP connection: after the data transmission is completed, the plant protection device sends a TCP FIN packet to the backend gateway, closes the TCP connection, and releases network resources. (6) Regular heartbeat check: to maintain the vitality of the connection and detect abnormal situations in a timely manner, the plant protection device can regularly send “heartbeat” detection packets to the backend gateway. This helps to ensure the stability of the connection and promptly detect and address any potential issues.

These data in table format are stored and managed using the Relational Database Management System (RDMS). The types of data mainly include String, Float, JPEG, Bool, and Date. In addition, the MinIO platform is used to achieve distribution, storage, and management of files. In addition, to improve computing resource management and service scheduling, various technologies, such as Spring MVC, Kubernetes, and Redis, are adopted. The optimal capacity of devices connected to this data structure is about 3800–5000 devices/single service.

#### 2.2.3 Service management layer

The service management layer contains various application servers for data processing and storage, making smart decisions, and, based on decisions, delivering the services over the network through protocols. Various analytical solutions can be applied to obtain intelligent decisions. The data storage server stores structured and unstructured data. The business server receives business requests from the application layer, performs specific operations in the database, and returns the results to the application layer. The database has Java+Tomcat+Mysql software architecture. Plant protection information is stored in two-dimensional structured data tables and associated data tables. A JDBC connection pool, MyBatis database middleware, and other technologies are used to realize efficient data query.

#### 2.2.4 Application layer

The application layer is the user interface that allows users to access requested services. Users can perform real-time monitoring of environmental data, remotely control plant protection equipment, manage online plant protection devices, and display data through visualization software, such as web pages and mobile apps. This layer also allows users to query various forms of historical data and export functions to conduct research and analysis based on historical data.

### 2.3 Plant protection devices

This section describes the design details and the components selected for the plant protection devices (hardware of the system), including the sensors and control circuit. In this study, two types of plant protection devices were designed, PPM-A and PPM-B, for use in facilities and fields, respectively.

#### 2.3.1 PPM-A

**Figure 3:**
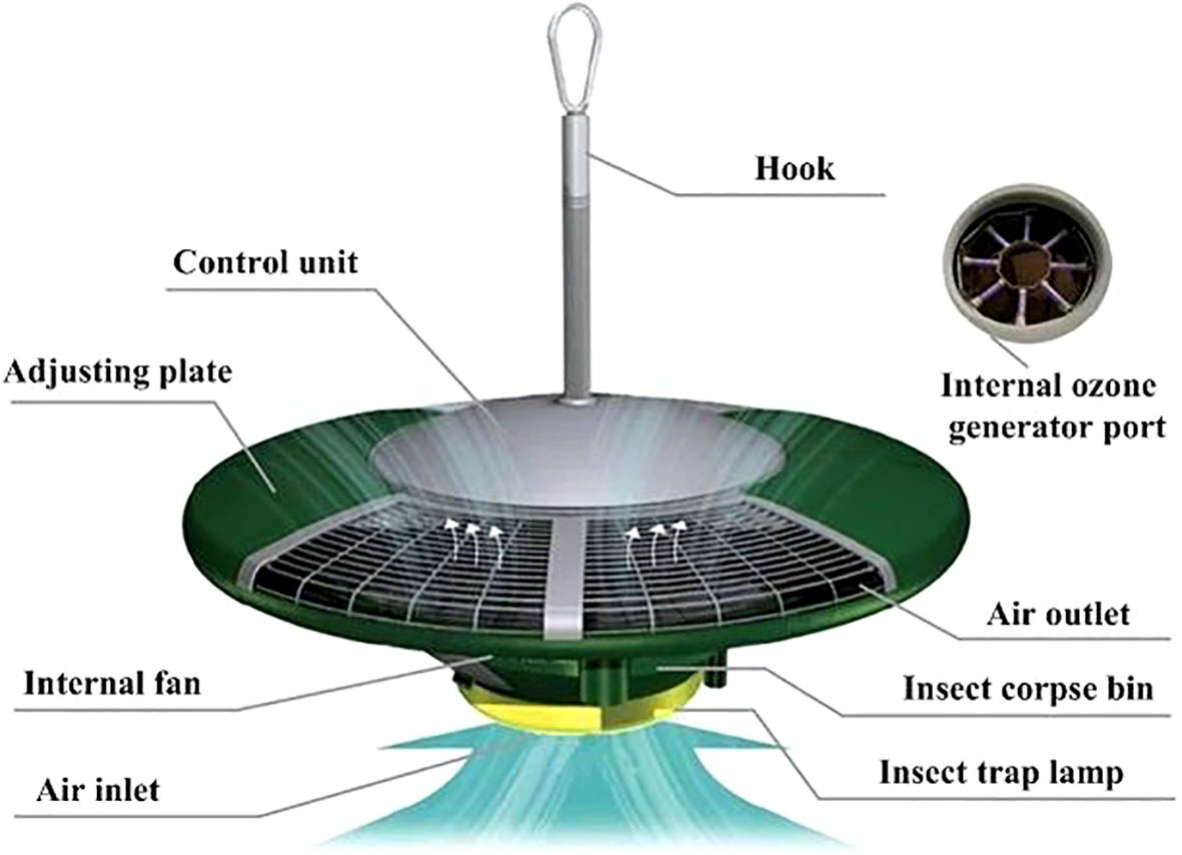
Structure of the facility plant protection device PPM-A.

PPM-A is a green prevention and control device for crop diseases and insect pests that has sterilization, disinsectization, auxiliary heating, and environmental data acquisition functions. The device includes an ozone generator, high-speed fan, trap lamp, adjusting plate, sensors, control device, and other modules (**Figure 3**). The specification parameters of PPM-A are as follows: diameter 800 mm; height 318 mm; boom length 410 mm; operating voltage 220 V, 50 Hz; rated power 290 W; ozone output 10 g/h; and ozone concentration at the air outlet 4.3–10.7 mg/m^3^.

In an indoor facility, PPM-A is suspended and installed at the top of a greenhouse. The device uses a high-voltage discharge method to produce ozone that is rapidly and evenly diffused into the whole facility space by a high-speed large-volume fan and special air duct. At the required concentration, the ozone oxidizes and decomposes eggs and larvae of insects, bacteria, and fungi, and inhibits virus reproduction. The device has yellow and blue lights to attract adult insects, which are then sucked into the device by the fan and killed.

#### 2.3.2 PPM-B

**Figure 4:**
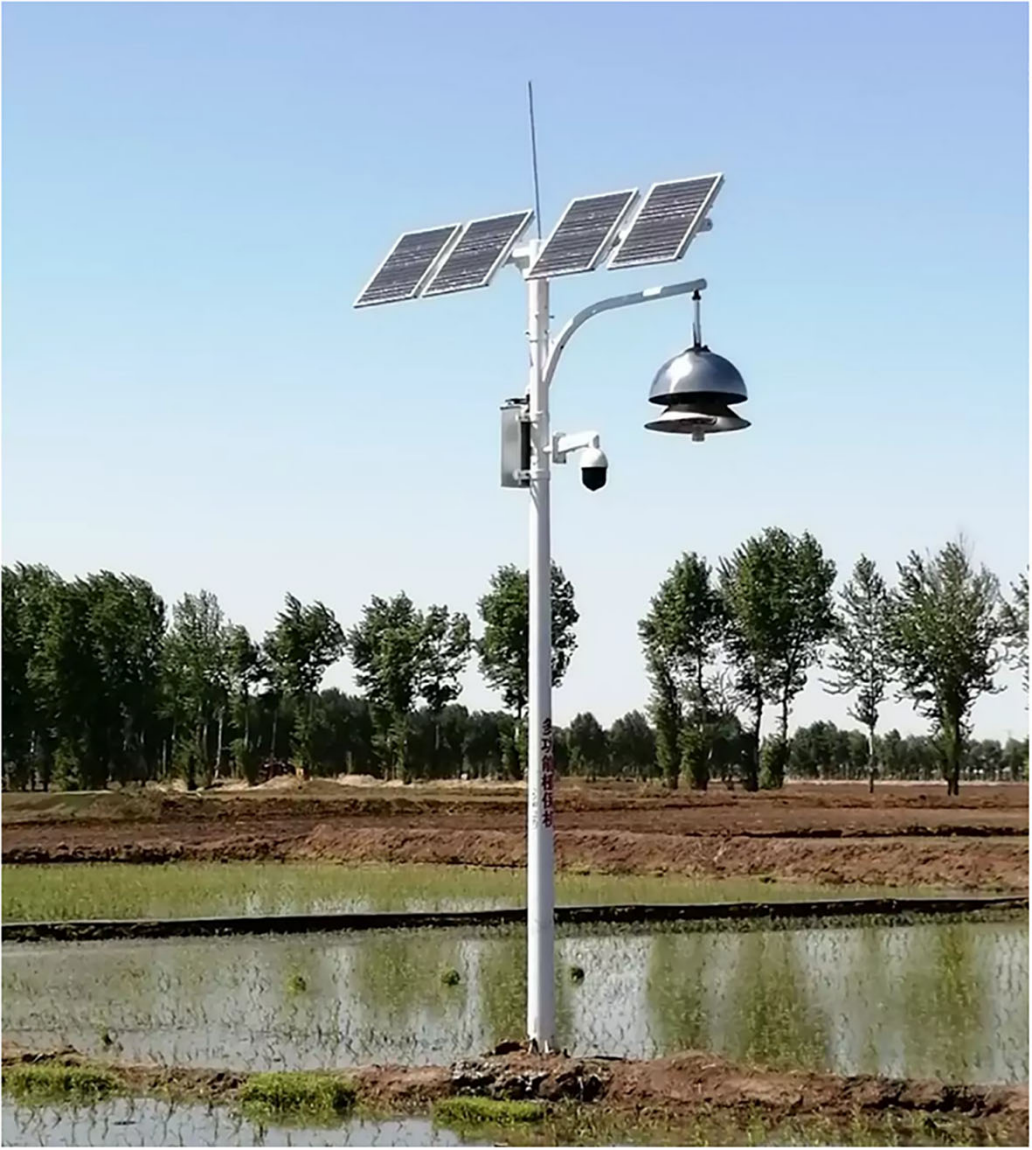
Structure of the field insecticidal plant protection device PPM-B.

PPM-B is an IoT-based solar insecticidal lamp. It has an insecticidal module, solar power supply module, control module, data transmission unit, surveillance camera, and sensors. The PPM-B structure is shown in **Figure 4**.

**Figure 5:**
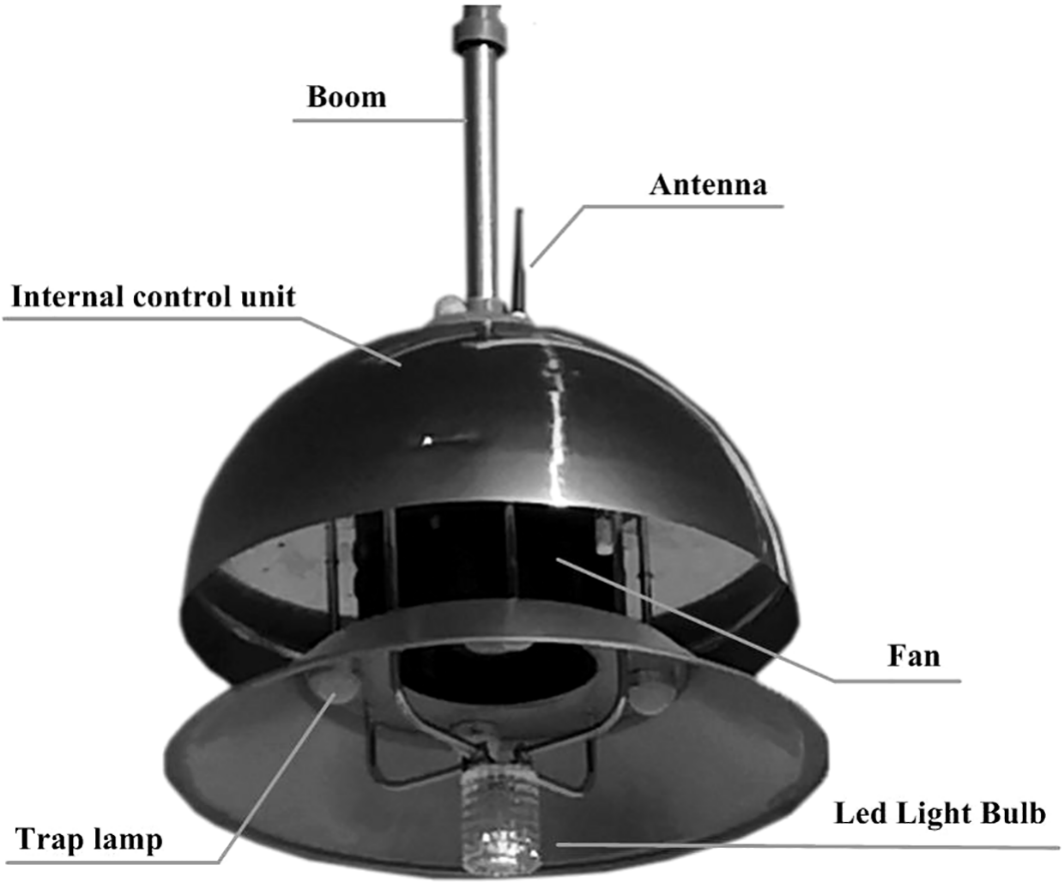
Structure of the insecticidal module of PPM-B.

The insecticidal module uses an LED lamp and characteristic spectrum to attract insects. The effective control radius of the insecticidal lamp is approximately 60 m. The module is also equipped with a high-speed large-volume fan with rated power 97 W, air volume 900 m^3^/h, and rotational speed 4600 1/min. The device has yellow and blue lights to attract adult insects, which are then sucked into the device by the fan and killed. The structure of the insecticidal module is shown in **Figure 5**. PPM-B does not cause environmental pollution and can be used to control lepidopteran pests in various field environments, such as farmland, vegetable gardens, and orchards.

The installed PPM-B sensors can monitor environmental data in the field in real time, including temperature, relative humidity, light intensity, and crop growth status, and promptly upload the collected data to the information management system through the wireless communication network. The device is equipped with solar panels and batteries that provide an uninterrupted power supply, ensuring continuous plant protection operations. Farmers can remotely control the fans and insect traps, adjust working modes, and monitor environmental data through a mobile app, and therefore do not need to operate the device on-site. A single account is sufficient to remotely control multiple devices with different numbers and to display device information, including device location, device number, base name, pest control frequency, device working status, and signal strength.

#### 2.3.3 Sensors

Various sensors can be deployed in the plant protection device for monitoring environmental changes. Technical characteristics of the sensors used in this study are listed in **Table 1**. These sensors, including the ambient light sensor (BH1750FVI), relative humidity and temperature sensor (SHT30), CO_2_ sensor (MG811), and other sensors, can communicate with a microcontroller embedded in the control circuit of the plant protection device through the I^2^C bus or RS485 interface. The microcontroller can monitor in real time environmental data, such as temperature, relative humidity, and light intensity, control the actuators for regulating the corresponding environment, and automatically upload these data to the information management system through the wireless transmission network.

#### 2.3.4 Control circuit

**Figure 6:**
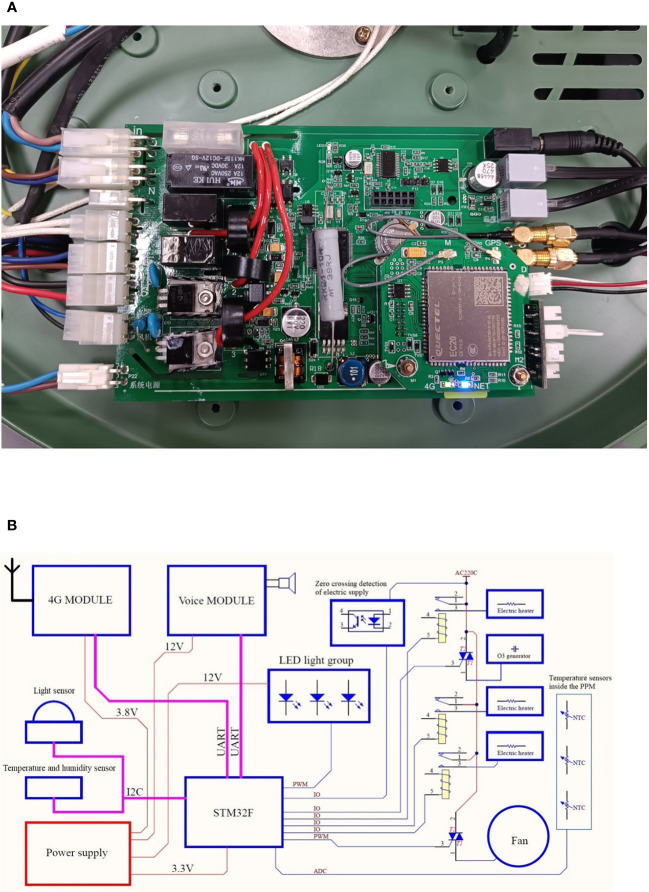
Control circuit used in the plant protection devices. **(A)** Control circuit implementation. **(B)** Block diagram of the hardware control circuit.

The control circuit of the plant protection device has four main modules: an ARM microprocessor, sensor interface, communication module, and device interface. All modules are controlled by an STM32F103VCT6 microprocessor. The control system converts the received information, such as temperature, relative humidity, and light intensity, into voltage or current signals through the sensor interfaces, transmits the data to the ARM microprocessor for processing, and controls the corresponding plant protection equipment to run based on the processing results. The control circuit used in the plant protection devices is shown in **Figure 6**.

Depending on the distance of data transmission, the control circuit can be equipped with wireless communication modules, such as GPRS, Wi-Fi, or Bluetooth, to achieve real-time data transmission. For long-distance data transmission, the STM32F103VCT6 microprocessor communicates with the EC20 wireless communication module through a serial port and sends the data to the server. The EC20 module is compatible with existing GSM/GPRS networks to ensure that it can work in remote areas that lack 3G and 4G networks. For short-distance data transmission, there are two options. One option is to use the control circuit to construct a Wi-Fi communication network by installing the ESP8266 module and send the data to the bound wireless router to achieve data transmission. The other option is to use the ATK-BLE01 Bluetooth module to realize real-time data transmission.

The plant protection equipment, including the ozone generation module, fan module, auxiliary heating module, trap lamp, and video monitoring module, can be controlled by the STM32F103VCT6 microprocessor with the device interface. The host computer is connected to the STM32F103VCT6 microprocessor through the wireless communication module. Depending on the growth status of the crops, farmers can send instructions through the host computer to achieve real-time control of the entire system.

### 2.4 Software of the system

The software of the IoT-based system consists of two elements: a web-based information management system and a mobile app. The web-based information management system interacts mainly with the data center to realize intelligent management of the plant protection data. The mobile app is used mainly for remote control and management of the hardware devices. In addition, the mobile app can interact with the data center to implement data query and visualization.

#### 2.4.1 Mobile app

A mobile app was designed to allow users to conveniently, accurately, and professionally operate the plant protection system to prevent and control diseases and insect pests. The system was developed on an Android platform using the Java language. The software packages used to develop the system were the Android Software Development Kit, Android Development Tools, and Eclipse, which were used as an integrated environment to develop the application in Java.

The main functional modules of the system were user registration, equipment management, equipment maintenance, and data query and visualization. The mobile app can be used to remotely control the plant protection device for real-time adjustment of the fan wind speed, ozone generation, environmental data collection, switching the trap lamp on and off, and display of device information. In addition, a single account can be used to control multiple devices in different locations for collaborative prevention and control of diseases and insect pests.

#### 2.4.2 Web-based information management system

The web-based information management system uses Browser/Server architecture to implement a data presentation service. This system has three main modules, namely, user management, equipment management, and plant protection data management. The user management module manages and maintains user information, such as the addition of user information, setting user permissions, and modification of user information. The equipment management module manages basic equipment information, equipment distribution, system operation logs, equipment maintenance records, and other information. The basic equipment information includes equipment number, installation location, user name, working mode, and equipment working status. The plant protection data management module interacts with the data center to manage plant protection data, such as query, export, visualization of graphs and tables, monitoring, and warning. The plant protection data include plant protection equipment, personnel information, environment information, and crop growth status. This plant protection data management module can also monitor plant protection data in real time. If anything abnormal is detected, an alarm with the relevant information is sent to the user’s mobile phone by SMS (short message service), so that the abnormal situation can be resolved as soon as possible. The module can also assist users to remotely monitor equipment for plant protection operations.

## 3 Results

This section sequentially describes the implementation of the IoT-based system, comprising software implementation, system application and environmental monitoring, and specifically its application in the prevention and control of crop diseases and insect pests.

### 3.1 System implementation and application

The developed plant protection system has been widely applied in China, where it has been used to control various diseases and insect pests of crops, such as rice, as well as in orchards and cucumber production facilities.

#### 3.1.1 Software implementation

**Figure 7:**
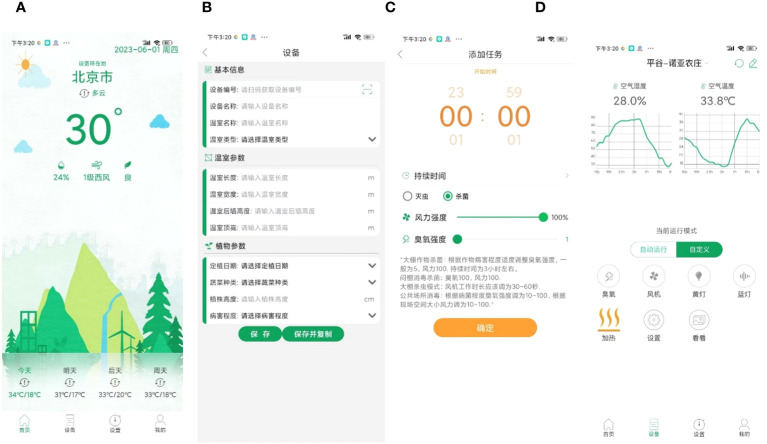
Representative example of the implementation of the mobile app. **(A)** Login interface, which displays the weather conditions in the area where the system is located. **(B)** Device registration interface, which has three main modules: basic equipment information, working environment, and crop growth information. Through this interface, users can register and use the plant protection equipment, as well as collect equipment working conditions and crop growth information. **(C)** Device operation mode interface, which allows users to set the ozone intensity, fan speed, and equipment working time for sterilization or pest control based on the working conditions and crop growth status. **(D)** Operating device interface, which displays environmental information on the equipment working area and device control buttons. Through this interface, users can remotely control and adjust the working mode of the device.

The mobile app can be used to remotely control the plant protection device for real-time adjustment of the fan wind speed, for ozone release, to collect environmental data, switch the trap lamp on and off, and other functions. A representative example of implementation of the mobile app is given in **Figure 7**.

**Figure 8:**
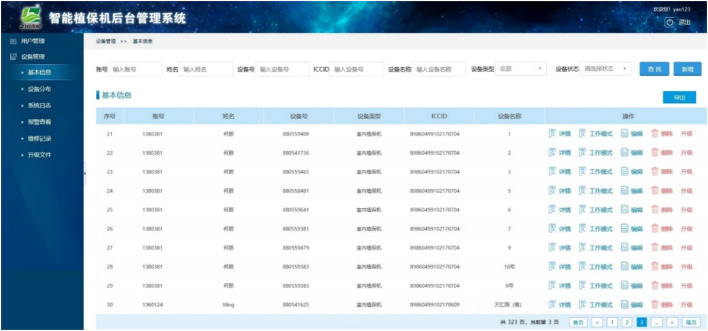
Representative example of the web-based information management system interface.

Representative operation results generated by the web-based information management system are shown in **Figure 8**. The web-based information management system interacts mainly with the data center to realize intelligent management of plant protection data. The system includes mainly user personal information, device hardware information, plant protection operation records, and environmental data, and manages the equipment to achieve high efficiency.

#### 3.1.2 System application

**Figure 9:**
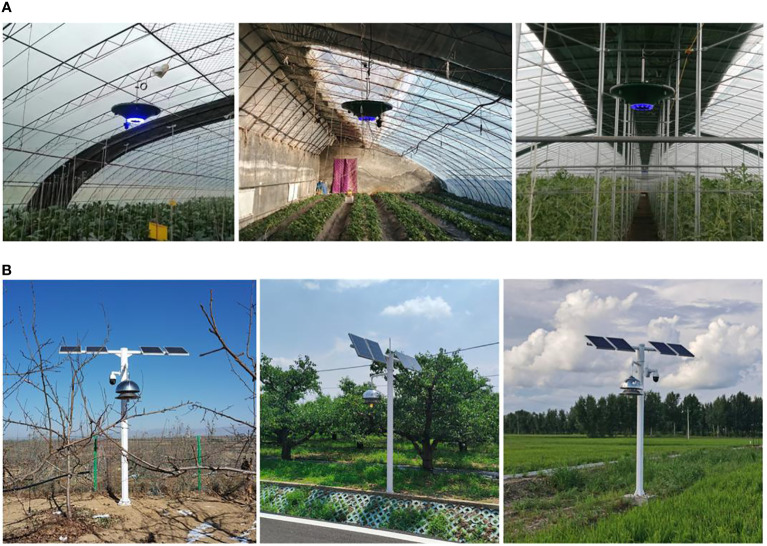
Actual application scenarios of plant protection devices. **(A)** Facility scenarios where PPM-A is used, **(B)** Field scenarios where PPM-B is used.

Actual application scenarios of plant protection devices are shown in **Figure 9**. The management of large-scale equipment applications and multi-device collaborative work can be achieved by this IoT-based system.

In this study, typical application scenarios of the system mainly include production facilities and fields, as shown in **Figure 9**. To achieve green prevention and control of diseases and insect pests, two types of plant protection devices were designed, PPM-A and PPM-B, for use in facilities and fields, respectively. A comparison of the system under different operation scenarios is as follows. For facility application scenarios, the system utilizes PPM-A for disease and insect pest control and environmental data collection. System operations include adjustment of ozone release, adjustment of the fan’s wind speed, opening of the trap lamp, auxiliary heating, and collection of environmental data, such as temperature, humidity, light intensity, and imagery. For field application scenarios, the system utilizes PPM-B for pest control and environmental data collection. System operations include adjustment of the fan’s wind speed, opening of the trap lamp, and collection of environmental data. Compared with the facility application scenarios, the ozone sterilization and auxiliary heating functions of the system cannot be used in field application scenarios.

In practical crop production, the installation and layout of plant protection equipment are mainly based on application scenarios and the needs for pest and disease prevention and control. After the launch of the devices, the optimal configuration for device operation can be achieved by users through the mobile app, as shown in **Figure 7**. For example, users can set the ozone intensity, fan speed, and equipment working time for sterilization or pest control based on the working conditions and crop growth status in the greenhouse. In addition, the user can use the web-based information management system to manage the collected plant protection data, as shown in **Figure 8**, such as the collected environmental data, device information, and personnel information. In general, the equipment needs to be powered on for 24 h during crop production. For functions such as insecticidal or ozone release, specific time settings must be set according to the growth of the crop. For example, ozone release can be selected to run once every 2 h at night.

#### 3.1.3 Environmental monitoring

**Figure 10:**
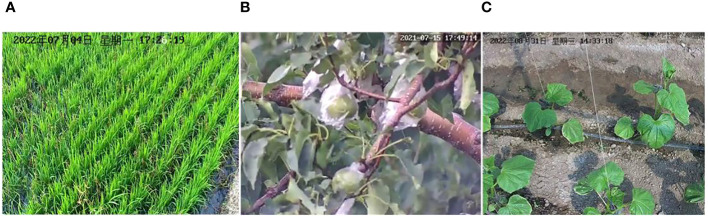
Images captured by the cameras deployed on the plant protection devices. **(A)** Rice in a field, **(B)** Apple tree in a field, and **(C)** Cucumber plants in a greenhouse.

**Figure 11:**
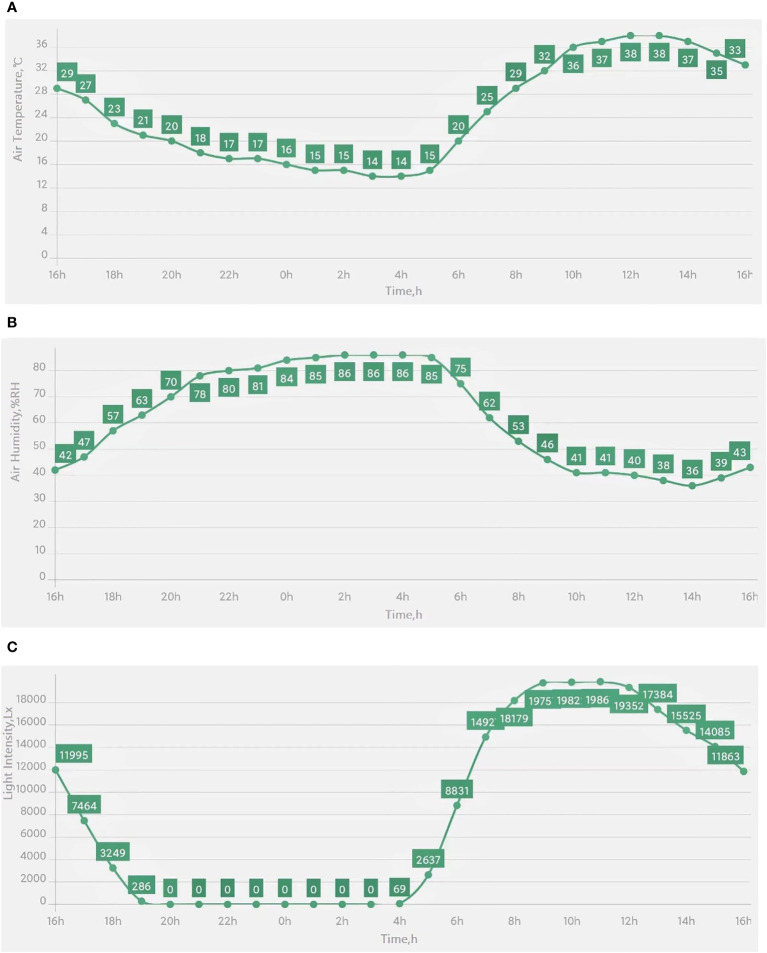
Environmental data collected by wireless sensors in the IoT-based system. **(A)** Air temperature (°C), **(B)** Air relative humidity (%RH), and **(C)** Light intensity (Lx).

Representative images captured by the cameras on plant protection devices are shown in **Figure 10**. Examples of the air temperature, relative humidity, and strength of illumination results collected by wireless sensors in the IoT-based system are given in **Figure 11**.

Such environmental data can be used by farmers through the mobile app to remotely monitor crops, determine the growth status and occurrence of diseases and pests in real time, and adjust the working mode of plant protection devices to achieve rapid and accurate prevention and control of crop diseases and pests. Crop production scenarios can also be monitored in real time to prevent the occurrence of abnormal events that can greatly reduce human resources. When the environmental parameters exceed the acceptable range, an alarm is generated and measures can be taken to mitigate the risk.

### 3.2 Effectiveness in prevention and control of crop diseases and insect pests

To verify the effectiveness of the developed plant protection system, disease and insect pest prevention and control experiments were conducted on different crops.

For the disease prevention and control experiments on facility crops, each disease prevention and control experiment was conducted separately. Three identical independent greenhouses were selected randomly for comparison. Three disease prevention and control methods were tested in sequence: release of ozone by the plant protection device, spray chemical pesticides, and no treatment. Other daily management measures, such as water and fertilizer application, were identical in the three facilities.

The plant protection device was suspended and installed at the top of a greenhouse, and was operated from 20:00 to 07:00 daily to avoid the impact of ozone on workers in the greenhouse. Users remotely controlled the working mode of the plant protection device and adjusted the ozone release amount and fan air volume in real time through the mobile app, based on the growth of the crops. For example, the ozone release rate was set to 15% and the fan wind intensity to 60%, and the device was operated continuously for 2 h at 2 h intervals.

A five-point sampling method was used to investigate the incidence of disease in crops in the greenhouses. All data were collected, organized, and summarized under the guidance of crop protection experts.

Two disease measures were used. The first was disease incidence, which was the number of diseased plants (or leaves) (*ND*) relative to the total number of plants (or leaves) (*NT*) in a plot. The second was disease index, which reflected the disease severity of plants in each plot and was calculated using the formula of Ma et al. (2017). The disease incidence (*M*) and disease index (*DI*) were calculated as follows (Equations 1, 2):


(1) $$\text{M}(\%)=\frac{N\text{D}}{N\text{T}}\times 100$$

(2) $$\text{DI}(\%)=\frac{\sum \left(\text{s}\times \text{n}\right)}{\text{NT}\times \text{S}}\;\times 100$$

where *s* and *n* are the disease severity grade and the number of plants in the grade, respectively, and *S* is the maximum disease severity grade.

Multiple sets of disease prevention and control experiments were conducted at different times, locations, and greenhouses. The performance of the three methods used for disease control is shown in **Table 2**. Data are shown as the mean ± SD. The mean percentage incidence of cucumber powdery mildew, tomato leaf mold, cowpea gray mold, celery early blight and gray mold, and cabbage damping-off in the greenhouse with the installed plant protection device was reduced by 36.68%, 12.26%, 16.63%, 6.4%, 9.6%, and 1.82%, respectively, compared with the incidence of these diseases in a conventionally managed greenhouse where no disease control measures were applied. Furthermore, the mean of disease incidence and disease index of these diseases in the greenhouse with ozone treatment was 10.51% and 2.32%, which is 1.54% and 1.05% lower, respectively, than that of the chemical pesticide treatment. These results showed that ozone effectively controlled cucumber powdery mildew, tomato leaf mold, cowpea gray mold, and celery early blight and gray mold, and had a small control effect on cabbage damping-off. Importantly, ozone had no adverse effects on the normal growth of the plants. The application of ozone as an air disinfectant can greatly reduce the use of chemical treatments, thereby reducing the production of harmful by-products associated with such treatments.

**Table 2:** Control of diseases by different methods, as indicated by the disease incidence (*M*; %) and disease index (*DI*; %), in vegetable crops grown in greenhouses.

Crop species	Disease	Our method (Ozone)		Chemical pesticides		No treatment	
		*M*	*DI*	*M*	*DI*	*M*	*DI*
Cucumber	Powdery mildew	28.33 ± 11.27	5.86 ± 2.54	35.55 ± 20.76	7.41 ± 5.51	65.01 ± 35.10	28.46 ± 22.82
Tomato	Leaf mold	5.18 ± 5.55	2.36 ± 2.35	5.99 ± 6.40	2.51 ± 2.36	17.44 ± 12.58	9.40 ± 7.13
Cowpea	Gray mold	9.24 ± 9.40	—	16.85 ± 11.17	—	25.87 ± 10.37	—
Celery	Early blight	2.13 ± 0.61	0.73 ± 0.15	3.73 ± 2.05	1.77 ± 1.06	8.53 ± 4.67	4.63 ± 2.71
	Gray mold	0.80 ± 0.80	0.33 ± 0.35	4.27 ± 2.66	1.77 ± 1.36	10.40 ± 3.20	5.70 ± 2.21
Cabbage	Damping-off	17.38 ± 15.88	—	5.89 ± 5.58	—	19.20 ± 16.01	—

**Figure 12:**
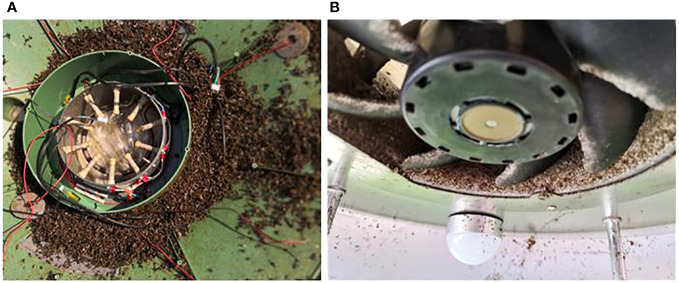
Trapping effects of the plant protection devices. **(A)** Adult pests killed by the high-speed rotating fans and high concentration of ozone in the cabin of PPM-A in a greenhouse. **(B)** Adult pests killed by the high-speed rotating fans of PPM-B in the field.

For the pest prevention and control experiments, visual images of trapped and killed pests were used to evaluate the insecticidal effects of the plant protection devices (**Figure 12**). This is because it was not possible to count the total number of pests in the facility and field scenarios, and accurately calculate the percentage of pests trapped and killed by the insecticidal lamps to evaluate the effectiveness of the lamps.

In the greenhouse, PPM-A has yellow and blue lights to attract adult insects, which are then sucked into the device and killed by the high-speed rotating fans and high concentration of ozone in the cabin of PPM-A (**Figure 12A**). In the field, PPM-B also has lights to attract adult insects, which are then sucked into the device by the fan and killed. Because of the high speed of the rotating fan in PPM-B, the corpses of pests are instantly crushed (**Figure 12B**). These results demonstrate the ability of the plant protection devices to effectively kill the pests, thereby reducing the amount of pesticides used in crop production and decreasing the costs of agrochemicals and labor.

In most scenarios, the designed plant protection device used yellow and blue lights to attract adult insects, which were then sucked into the device and killed by the high-speed rotating fans. Thus, the developed device mainly exerted satisfactory control of adult insects through phototaxis. At present, it can effectively kill more than 30 types of insect pests, such as *Spodoptera litura*, *Spodoptera exigua*, *Spodoptera frugiperda*, whiteflies, *Nilaparavata lugens*, and *Liriomyza sativae*.

## 4 Discussion

### 4.1 Comparison and advantages

With the rapid development of information and communication technologies, smart plant protection is gradually becoming a novel research focus (Huang and Shu, 2021; Nayagam et al., 2023). The approach has the advantages of being sustainable, intelligent, and unmanned. In the present study, we integrated ozone sterilization and light-trap technologies to develop a plant protection system for the prevention and control of crop diseases and insect pests. The experimental results, as shown in **Table 2** and **Figure 12**, demonstrated that the proposed method had favorable control effects on crop diseases and insect pests. This system has the following advantages over traditional methods that use chemical pesticides. 1) The ozone sterilization and light-trap technologies avoid potential problems, such as accumulation of pesticide residues, environmental pollution, and disease and insect resistance, caused by excessive use of chemical pesticides. 2) The system can be operated at any time according to needs, whereas, to use chemical pesticides, factors such as pesticide dosage and pesticide variety, and spraying date and pattern, need to be considered. For example, different types of chemical pesticides and dosages were applied to control cucumber powdery mildew and tomato gray mold. 3) The system can be used continuously for a long time; indeed, some of our systems can be used for more than 10 years. These systems have effectively reduced the cost of production inputs, such as pesticide purchase and labor costs for spraying pesticides. Our research results indicate that the costs of agrochemicals and labor can be decreased by approximately 22,500 yuan per hm^2^ during the crop growing period. 4) Plant protection devices can be installed at multiple sites simultaneously using the information management system to standardize control of crop diseases and pests. The various spraying dates, dosages, and spraying patterns required for chemical pesticides make simultaneous implementation difficult. 5) The system can realize real-time collection and monitoring of environmental data. The information generated can be used by farmers to determine the growth status of crops and occurrence of diseases and pests in real time, and adjust the working mode of the plant protection devices to achieve rapid and accurate prevention and control of crop diseases and pests. In addition, the collected real-time environmental data are valuable for farmers to monitor conditions that favor the proliferation of crop diseases, and to implement precautionary measures before infection/infestation is severe. Therefore, the incidence of serious diseases will be greatly reduced. In the worst case that disease severity is acute, even pesticides may struggle to be effective. The system will record the environmental data coincident with the occurrence of the disease to avoid recurrence in the future. Overall, the operation of the developed plant protection system is more convenient and has superior usability compared with the spray application of chemical pesticides.

Ozone sterilization and light traps have been widely applied in the prevention and control of diseases and pests. In recent years, with the development and application of agricultural IoT, the above-mentioned technologies will be more convenient to use in actual production. However, some limitations remain, as discussed in Section 1. Previous similar systems are costly, incorporate online–offline modes of data collection, and do not fulfill the rapid requirements of a user-friendly interface. To overcome these difficulties, the advantages of ozone sterilization, light trap, and IoT technologies were combined and a system developed for the prevention and control of crop diseases and insect pests. The following characteristics enhance the effectiveness of this system. To be suitable for both facility- and field-based production scenarios, two types of plant protection devices were designed: one suitable for production facilities and one for field use. The system can achieve efficient management of large-scale equipment applications and multi-device collaborative work to prevent and control pests and diseases. Moreover, the system has a mobile device app. The mobile app provides farmers with remote control of the plant protection devices and enables regulation of the working mode of the plant protection devices in real time, for example, adjustment of insecticidal lamp operation hours or ozone release. This system not only ensures standardization and informatization of plant protection operations, but also allows for dynamic adjustments in coordination with crop growth. In short, compared with other similar systems, our system exhibits stability, feasibility, and ease of scalability in production facilities and in fields. In particular, the system is user-friendly, portable, intelligent and inexpensive. Compared with similar systems summarized in **Table 3**, the novelty of the present system lies in three aspects: technology integration, data generation, and practical applications.

**Table 3:** Comparison of relevant operational systems for prevention and control of diseases and insect pests in agriculture.

Reference	Function description	Control model	Data acquisition	Device condition monitoring	Prevention and control targets	Scenario
(Lam et al., 2013)	Designed a brown planthopper surveillance network	Manual setup	Real-time data acquisition	N/A	Insect pests	Farmlands
(Wang et al., 2023)	Established an efficient light trapping and control technology for insect pests in peanut fields	Manual setup and time control	Offline modes of data collection	No	Insect pests	Farmlands
(Zhang and Xing, 2014)	Developed an intelligent insecticidal lamp	Manual setup and time control	Offline modes of data collection	No	Insect pests	Farmlands
(Khattab et al., 2019)	Designed a monitoring system for early plant disease forecast	Manual setup	Real-time data acquisition	N/A	Diseases	Farmlands
(Carvalho et al., 2021)	Designed a smart LED lamp to monitor insect populations	Manual setup and time control	N/A	N/A	Insect pests	Farmlands
(Nayagam et al., 2023)	Proposed a parallel and distributed simulation framework for pest management and agricultural monitoring tools	N/A	Real-time data acquisition	N/A	Insect pests	Farmlands
The present study	Established an unified system of prevention and control for crop diseases and insect pests	Remote control using mobile app	Real-time data acquisition	Remote monitoring using mobile app	Diseases and insect pests	Farmlands, greenhouses

“N/A” indicates that the information was not described in the publication, or is not clear whether it monitors specific data.

#### 4.1.1 Technology integration

In the field of plant protection, we combined ozone sterilization, light trap, and IoT technologies, and developed a novel green prevention and control technology for crop diseases and insect pests to reduce the amounts of chemical pesticides used and to promote sustainable crop production. The proposed system has two main components: a plant protection device (the hardware) and an information management system (the software). To the best of our knowledge, the proposed system is the first green prevention and control system of diseases and insect pests that integrates multiple functions, such as ozone sterilization, disinsectization, remote control of crop protection equipment, data collection and management, and can simultaneously cover application scenarios such as production facilities and fields. Although currently various agricultural IoT systems have been developed (Tzounis et al., 2017; Khanna and Kaur, 2020; Gaikwad et al., 2021; Tashakkori et al., 2021; Nayagam et al., 2023), they are rarely applied in the field of plant protection, and existing systems focus mostly on data collection and management, lacking support for plant protection devices. These systems are difficult to integrate with plant protection devices to achieve green prevention and control of pests and diseases in different actual production.

#### 4.1.2 Data generation

The proposed system can use plant protection equipment to collect various environmental data in real time (as shown in **Figures 10**, **11**), such as temperature, humidity, light intensity, and crop images. Farmers can remotely monitor a crop through the mobile app, determine the growth status and occurrence of diseases in real time, and adjust the working mode of the plant protection devices to achieve rapid and accurate prevention and control of crop diseases. In addition, the collected real-time environmental data are valuable for the subsequent development of intelligent plant protection devices and systems. However, currently used plant protection devices often lack the necessary data display boards. Moreover, earlier systems are costly and are not equipped with data management dashboard and mobile applications. Thus, farmers find it difficult to obtain real-time data on plant protection operations.

#### 4.1.3 Practical applications

The system has been in use for more than 3 years in China. Actual application scenarios are shown in **Figure 9**. The system effectively assists farmers in plant protection management for crop production. The system can be operated on a daily or hourly basis, depending on the users’ needs. The system not only greatly reduces the amount of chemical pesticides used, and decreases the associated costs of agrochemicals and labor (approximately 22,500 yuan/hm^2^), but also can be applied to unify the scheduling of plant protection operations and multi-device collaborative work, thereby improving the usage efficiency of the plant protection devices and managing multiple crop production scenarios. In addition, the system exhibits stability, feasibility, and ease of scalability in production facilities and in fields. In particular, the system is user-friendly, portable, intelligent, and inexpensive. Moreover, farmers are not required to be on-site for inspection and avoid a series of problems caused by spraying chemical pesticides. Previous similar systems mainly focused on experimental applications and few have been engaged in actual production large-area promotion and applications.

### 4.2 Limitations and prospects

Intelligence and precision are important research avenues for intelligent plant protection. In practical applications, intelligent control of operational equipment has not yet been fully achieved. Here, some of the present study’s limitations are mentioned, together with some improvements that could be made to the proposed system to further refine it.

First, accurate and rapid classification of diseases is an important basis for early disease monitoring, diagnostics, and prevention (Abdullah et al., 2023; Liu and Wang, 2023). In recent years, deep convolutional neural networks have achieved remarkable success in disease image classification (Ning et al., 2023; Zhang et al., 2023). In our research team’s previous work, several image classification methods have been proposed (Wang et al., 2022b; Wei et al., 2022). These methods have not been incorporated in the designed system because the present study mainly focused on the development and integration of plant protection systems. Thus, when utilizing the system, adjustment of the working mode of a plant protection device is mainly based on the user’s personal experience or the guidance of technical personnel, which can easily lead to untimely and inaccurate control of pests and diseases. In future work, we plan to combine these methods with the present system to realize accurate disease identification and integration with the intelligent plant protection devices based on the classification results.

Second, adaptive adjustment of the ozone concentration based on the growth period of crops and disease severity has not been implemented. In addition, using the same ozone concentration to control different types of diseases in different crops is suboptimal. For example, the ozone concentration required to effectively control cucumber powdery mildew and other air-borne diseases does not control cabbage damping-off and other typical soil-borne diseases, as shown in **Table 2**. Although the proposed method can achieve standardization and normalization of ozone use, intelligent ozone control technology is still lacking. Implementation of intelligent detection, image processing, and artificial intelligence methods in the system to achieve accurate, adaptive, and closed-loop control will be a focus of future work. We aim to develop algorithmic models to maximize the use of real-time incidence data for pests and diseases to enable the system to achieve rapid and precise prevention and control of pests and diseases under diverse application scenarios.

Third, the technology for automatic adjustment of the operating hours of the developed PPM-B (solar insecticidal lamps) is still lacking. In practical applications, PPM-B cannot intelligently adjust the operating hours based on information, such as pest density and activity time, in different seasons and regions, which limits the effectiveness and efficiency of the devices. Although, in this study, users can use the mobile app to dynamically adjust the operating hours of the plant protection devices, which to some extent alleviates the above-mentioned problems and improves the efficiency of plant protection device usage, the strategy for adjustment of the operating hours of the insecticidal lamps is still based on user personal experience and actual observation. Therefore, the technology for dynamic adjustment of the operating hours of the insecticidal lamps is an important focus for future research. To date, multiple research groups have conducted in-depth research on the intelligent control of insecticidal lamps and proposed intelligent control algorithms (Yang et al., 2021; Shao et al., 2022; Yang et al., 2023). In future research, we will integrate these research results into the developed system to achieve automatic adjustment and intelligent control of the operating hours of the insecticidal lamp. For example, the operating hours of the insecticidal lamp could be adjusted based on the phototaxis rhythm of pests (Yao et al., 2023).

In addition, direct performance indicators, such as disease incidence and visual images of killed pests, were used to evaluate the performance of the system. However, indirect performance indicators, such as crop yield, economic impact, and the ecological environment, were not used. This is because these indicators require more systematic and long-term tracking research and experimentation. In future work, we will conduct experiments to evaluate these metrics.

## 5 Conclusions

We developed a system for prevention and control of crop diseases and insect pests by combining ozone sterilization, light trap, and IoT technologies. Two types of plant protection devices were designed, one for use in production facilities and one for use in fields, which removed the need to apply chemical pesticides during crop production. These devices were equipped with various sensors that collect and monitor real-time environmental data that can be used by farmers to determine the growth status of crops and improve the efficiency of crop production. The IoT-based information management system can be used remotely to control the plant protection devices and intelligently manage the collected plant protection data. Moreover, this system can perform unified scheduling of plant protection operations and multi-device collaborative work. We showed that the system is convenient to operate and exhibits stability, feasibility, and strong scalability in facilities and in fields. The system controls diseases and pests effectively with no adverse effects on the normal growth of the crop, and greatly reduces the amount of chemical pesticides used, which significantly decreases the costs of agrochemicals and labor. This work provides a reference for development of intelligent equipment for the prevention and control of diseases and insect pests using physical control methods.

The next challenge is to achieve intelligent and precise prevention and control of diseases and pests based on incidence data. In future work, we will focus on the precise control of ozone concentration, adaptive adjustment of insecticidal lamp operation hours, and real-time identification and monitoring of diseases and pests, with the aim of developing an intelligent version of the present system.

## Data availability statement

The original contributions presented in the study are included in the article/supplementary material. Further inquiries can be directed to the corresponding authors.

## Author contributions

ZW: Conceptualization, Methodology, Software, Validation, Writing – original draft. XQ: Project administration, Writing – review & editing. YW: Data curation, Investigation, Visualization, Writing – original draft. HY: Data curation, Investigation, Visualization, Writing – original draft. CM: Formal analysis, Software, Writing – review & editing.

## References

[B1] Abdelfattah N. A. H. Al-Qahtani A. R. Qari S. H. (2021). Scot-marker analysis of Oryzaephilus surinamensis L. (Coleoptera: Silvanidae) and stored date kernels of phoenix dactylifera (L.) fumigated with ozone and phosphine gases. J. Asia-Pac. Entomol. 24, 843–849. doi: 10.1016/j.aspen.2021.07.009

[B2] Abdullah H. M. Mohana N. T. Khan B. M. Ahmed S. M. Hossain M. Islam K. S. . (2023). Present and future scopes and challenges of plant pest and disease (p&d) monitoring: remote sensing, image processing, and artificial intelligence perspectives. Remote Sens. Applications: Soc. Environ. 32, 100996. doi: 10.1016/j.rsase.2023.100996

[B3] Alimohammadi M. Naderi M. (2021). Effectiveness of ozone gas on airborne virus inactivation in enclosed spaces: a review study. Ozone-Sci. Eng. 43, 21–31. doi: 10.1080/01919512.2020.1822149

[B4] Bian L. Cai X. Luo Z. Li Z. Chen Z. (2018). Decreased capture of natural enemies of pests in light traps with light-emitting diode technology. Ann. Appl. Biol. 173, 251–260. doi: 10.1111/aab.12458

[B5] Carvajal-Yepes M. Cardwell K. Nelson A. Garrett K. A. Giovani B. Saunders D. G. O. . (2019). A global surveillance system for crop diseases. Sci. (American Assoc. Advancement Science) 364, 1237–1239. doi: 10.1126/science.aaw1572 31249049

[B6] Carvalho M. W. M. D. Hickel E. R. Bertoldi B. Knabben G. C. Novaes Y. R. D. (2021). Design of a smart led lamp to monitor insect populations in an integrated pest management approach. Rev. Bras. Engenharia Agrícola E Ambiental 25, 270–276. doi: 10.1590/1807-1929/agriambi.v25n4p270-276

[B7] Deutsch C. A. Tewksbury J. J. Tigchelaar M. Battisti D. S. Merrill S. C. Huey R. B. . (2018). Increase in crop losses to insect pests in a warming climate. Sci. (American Assoc. Advancement Science) 361, 916–919. doi: 10.1126/science.aat3466 30166490

[B8] Díaz-López M. Siles J. A. Ros C. Bastida F. Nicolás E. (2022). The effects of ozone treatments on the agro-physiological parameters of tomato plants and the soil microbial community. Sci. Total Environ. 812, 151429. doi: 10.1016/j.scitotenv.2021.151429 34742984

[B9] Ebihara K. Mitsugi F. Ikegami T. Nakamura N. Hashimoto Y. Yamashita Y. . (2013). Ozone-mist spray sterilization for pest control in agricultural management. Eur. Phys. J. Appl. Phys. 61, 24318. doi: 10.1051/epjap/2012120420

[B10] Epelle E. I. Macfarlane A. Cusack M. Burns A. Thissera B. Mackay W. . (2022). Bacterial and fungal disinfection via ozonation in air. J. Microbiol. Methods 194, 106431. doi: 10.1016/j.mimet.2022.106431 35131364

[B11] Erler F. Bayram Y. (2021). Efficacy of mass trapping of tomato moth, tuta absoluta (meyrick 1917) (lepidoptera: gelechiidae), using a new-designed light trap in reducing leaf and fruit damages in greenhouse-grown tomatoes. J. Plant Dis. Prot. 128, 1177–1185. doi: 10.1007/s41348-021-00473-8

[B12] Fernández I. A. L. Monje-Ramirez I. de Velásquez M. T. O. L. (2019). Tomato crop improvement using ozone disinfection of irrigation water. Ozone-Sci. Eng. 41, 398–403. doi: 10.1080/01919512.2018.1549474

[B13] Foroughi M. Khiadani M. Kakhki S. Kholghi V. Naderi K. Yektay S. (2022). Effect of ozonation-based disinfection methods on the removal of antibiotic resistant bacteria and resistance genes (arb/args) in water and wastewater treatment: a systematic review. Sci. Total Environ. 811, 151404. doi: 10.1016/j.scitotenv.2021.151404 34767893

[B14] Fujiwara K. Fujii T. (2002). Effects of spraying ozonated water on the severity of powdery mildew infection on cucumber leaves. Ozone-Sci. Eng. 24, 463–469. doi: 10.1080/01919510208901635

[B15] Gaikwad S. V. Vibhute A. D. Kale K. V. Mehrotra S. C. (2021). An innovative iot based system for precision farming. Comput. Electron. Agric. 187, 106291. doi: 10.1016/j.compag.2021.106291

[B16] Gao D. Sun Q. Hu B. Zhang S. (2020). A framework for agricultural pest and disease monitoring based on internet-of-things and unmanned aerial vehicles. Sensors 20, 1487. doi: 10.3390/s20051487 32182732 PMC7085563

[B17] Han H. Zhang J. Liu M. Zhao F. Wang G. Lü Y. (2020). The application of fan suction solar energy insecticidal lamp in fresh corn fields and its effect on the biodiversity of arthropods. Acta Phytophylacica Sin. 47, 1234–1243. doi: 10.13802/j.cnki.zwbhxb.2020.2020069

[B18] Hirschi M. Stoeckli S. Dubrovsky M. Spirig C. Calanca P. Rotach M. W. . (2012). Downscaling climate change scenarios for apple pest and disease modeling in Switzerland. Earth Syst. Dynam. 3, 33–47. doi: 10.5194/esd-3-33-2012

[B19] Huang K. Shu L. (2021). Grand challenges in sustainable and intelligent phytoprotection. Front. Plant Sci. 12. doi: 10.3389/fpls.2021.755510 PMC8635507 34868144

[B20] Isikber A. A. Athanassiou C. G. (2015). The use of ozone gas for the control of insects and micro-organisms in stored products. J. Stored Prod. Res. 64, 139–145. doi: 10.1016/j.jspr.2014.06.006

[B21] Kagan C. R. Arnold D. P. Cappelleri D. J. Keske C. M. Turner K. T. (2022). Special report: the internet of things for precision agriculture (iot4ag). Comput. Electron. Agric. 196, 106742. doi: 10.1016/j.compag.2022.106742

[B22] Karmakar S. Das P. Panda D. Xie K. Baig M. J. Molla K. A. (2022). A detailed landscape of crispr-cas-mediated plant disease and pest management. Plant Sci. 323, 111376. doi: 10.1016/j.plantsci.2022.111376 35835393

[B23] Katsoulas N. Antoniadis D. Nikitas A. (2021). A web-based system for fungus disease risk assessment in greenhouses: system development. Comput. Electron. Agric. 188, 106326. doi: 10.1016/j.compag.2021.106326

[B24] Khanna A. Kaur S. (2020). Internet of things (iot), applications and challenges: a comprehensive review. Wirel. Pers. Commun. 114, 1687–1762. doi: 10.1007/s11277-020-07446-4

[B25] Khattab A. Habib S. E. D. Ismail H. Zayan S. Fahmy Y. Khairya M. M. (2019). An iot-based cognitive monitoring system for early plant disease forecast. Comput. Electron. Agric. 166, 105028. doi: 10.1016/j.compag.2019.105028

[B26] Lam H. B. Phan T. T. Vuong L. H. Huynh H. X. Pottier B. (2013). Designing a brown planthoppers surveillance network based on wireless sensor network approach. Comput. Sci., 1–6. doi: 10.48550/arXiv.1312.3692

[B27] Li K. L. Shu L. Huang K. Sun Y. Yang F. Zhang Y. . (2019). Research and prospect of solar insecticidal lamps internet of things. Smart Agric. 1, 13–28. doi: 10.12133/j.smartag.2019.1.3.201905-SA001

[B28] Liu Y. Liu C. Zhang J. C. Zhao S. Y. (2013). Discussion on applicability of the technology of using light to trap in the field of pests and diseases control in tea plantation of China. Key Eng. Materials 575-576, 487–493. doi: 10.4028/www.scientific.net/KEM.575-576.487

[B29] Liu J. Wang X. (2023). Tomato disease object detection method combining prior knowledge attention mechanism and multiscale features. Front. Plant Sci. 14. doi: 10.3389/fpls.2023.1255119 PMC10590886 37877077

[B30] Ma Y. Li Y. Lai H. Guo Q. Xue Q. (2017). Effects of two strains of streptomyces on root-zone microbes and nematodes for biocontrol of root-knot nematode disease in tomato. Appl. Soil Ecol. 112, 34–41. doi: 10.1016/j.apsoil.2017.01.004

[B31] Mack G. Finger R. Ammann J. El Benni N. (2023). Modelling policies towards pesticide-free agricultural production systems. Agric. Syst. 207, 103642. doi: 10.1016/j.agsy.2023.103642

[B32] Morrison C. M. Hogard S. Pearce R. Gerrity D. von Gunten U. Wert E. C. (2022). Ozone disinfection of waterborne pathogens and their surrogates: a critical review. Water Res. 214, 118206. doi: 10.1016/j.watres.2022.118206 35276607

[B33] Nayagam M. G. Vijayalakshmi B. Somasundaram K. Mukunthan M. A. Yogaraja C. A. Partheeban P. (2023). Control of pests and diseases in plants using iot technology. Measurement: Sensors 26, 100713. doi: 10.1016/j.measen.2023.100713

[B34] Nielsen A. L. Holmstrom K. Hamilton G. C. Cambridge J. Ingerson-Mahar J. (2013). Use of black light traps to monitor the abundance, spread, and flight behavior of halyomorpha halys (hemiptera: pentatomidae). J. Econ. Entomol. 106, 1495–1502. doi: 10.1603/EC12472 23865219

[B35] Ning H. Liu S. Zhu Q. Zhou T. (2023). Convolutional neural network in rice disease recognition: accuracy, speed and lightweight. Front. Plant Sci. 14. doi: 10.3389/fpls.2023.1269371 PMC10646333 38023901

[B36] Pandiselvam R. Kaavya R. Jayanath Y. Veenuttranon K. Lueprasitsakul P. Divya V. . (2020). Ozone as a novel emerging technology for the dissipation of pesticide residues in foods–a review. Trends Food Sci. Technol. 97, 38–54. doi: 10.1016/j.tifs.2019.12.017

[B37] Pandiselvam R. Subhashini S. Priya E. P. B. Kothakota A. Ramesh S. V. Shahir S. (2019). Ozone based food preservation: a promising green technology for enhanced food safety. Ozone-Sci. Eng. 41, 17–34. doi: 10.1080/01919512.2018.1490636

[B38] Qiao X. Jia H. Wang C. Wang K. Yan B. Guo W. (2020). Design and experiment of ozone sterilizer device for organic matrix. Trans. Chin. Soc. Agric. Machinery 51, 138–145. doi: 10.6041/j.issn.1000-1298.2020.07.016

[B39] Sang W. Gao Q. Zhang C. Huang Q. Lei C. Wang X. (2022). Researches and applications of physical control of agricultural insect pests in China. Acta Phytophylacica Sin. 49, 173–183. doi: 10.13802/j.cnki.zwbhxb.2022.2022813

[B40] Sayem N. S. Chowdhury S. Haque A. H. M. O. Ali M. R. Alam M. S. Ahamed S. . (2023). Iot-based smart protection system to address agro-farm security challenges in Bangladesh. Smart Agric. Technol. 6, 100358. doi: 10.1016/j.atech.2023.100358

[B41] Schreinemachers P. Grovermann C. Praneetvatakul S. Heng P. Nguyen T. T. L. Buntong B. . (2020). How much is too much? Quantifying pesticide overuse in vegetable production in southeast asia. J. Clean Prod. 244, 118738. doi: 10.1016/j.jclepro.2019.118738

[B42] Shao S. Zhang Q. Guo S. Sun L. Qiu X. Meng L. (2022). Intelligent farm meets edge computing: energy-efficient solar insecticidal lamp management. IEEE Syst. J. 16, 3668–3678. doi: 10.1109/JSYST.2022.3174925

[B43] Singh V. K. Singh R. Kumar A. Bhadouria R. (2021). Chapter 2 - current status of plant diseases and food security. Eds. Kumar A. Droby S. (Duxford: Woodhead Publishing), 19–35. doi: 10.1016/B978-0-12-821843-3.00019-2

[B44] Song B. Seiber J. N. Duke S. O. Li Q. X. (2020). Green plant protection innovation: challenges and perspectives. Engineering 6, 483–484. doi: 10.1016/j.eng.2020.04.001 32300489 PMC7159853

[B45] Szumigaj-Tarnowska J. Szafranek P. Uliński Z. Ślusarski C. (2020). Efficiency of gaseous ozone in disinfection of mushroom growing rooms. J. Hortic. Res. 28, 91–100. doi: 10.2478/johr-2020-0017

[B46] Takayama M. Ebihara K. Stryczewska H. Ikegami T. Gyoutoku Y. Kubo K. . (2006). Ozone generation by dielectric barrier discharge for soil sterilization. Thin Solid Films 506, 396–399. doi: 10.1016/j.tsf.2005.08.332

[B47] Tashakkori R. Hamza A. S. Crawford M. B. (2021). Beemon: an iot-based beehive monitoring system. Comput. Electron. Agric. 190, 106427. doi: 10.1016/j.compag.2021.106427

[B48] Terence S. Purushothaman G. (2020). Systematic review of internet of things in smart farming. Trans. Emerg. Telecommun. Technol. 31, e3958. doi: 10.1002/ett.3958

[B49] Tu H. Tang N. Hu X. Yao Z. Wang G. Wei H. (2016). Led multispectral circulation solar insecticidal lamp application in rice field. Trans. Chin. Soc. Agric. Eng. 32, 193–197. doi: 10.11975/j.issn.1002-6819.2016.16.027

[B50] Tzounis A. Katsoulas N. Bartzanas T. Kittas C. (2017). Internet of things in agriculture, recent advances and future challenges. Biosyst. Eng. 164, 31–48. doi: 10.1016/j.biosystemseng.2017.09.007

[B51] Vincent C. Weintraub P. Hallman G. (2009). Chapter 200 - physical control of insect pests. Eds. Resh V. H. Cardé R. T. (San Diego: Academic Press), 794–798. doi: 10.1016/B978-0-12-374144-8.00209-5

[B52] Wang Y. Qiao X. Wang Z. (2022a). Application of ozone treatment in agriculture and food industry. A review. Inmateh - Agric. Eng. 3, 861–872. doi: 10.35633/inmateh-68-86

[B53] Wang Z. Wang K. Wang X. Pan S. Qiao X. (2022b). Dynamic ensemble selection of convolutional neural networks and its application in flower classification. Int. J. Agric. Biol. Eng. 15, 216–223. doi: 10.25165/j.ijabe.20221501.6313

[B54] Wang H. Yan K. Liu T. Kuang C. Cai F. Wang X. . (2023). Trapping effects of solar light traps on insects in peanut fields. J. Plant Prot. 50, 136–145. doi: 10.13802/j.cnki.zwbhxb.2021.2021111

[B55] Wei Y. Wang Z. Qiao X. Zhao C. (2022). Lightweight rice disease identification method based on attention mechanism and efficientnet. J. Chin. Agric. Mechanization 43, 172–181. doi: 10.13733/j.jcam.issn.2095-5553.2022.11.024

[B56] Yang X. Shu L. Li K. Huo Z. Shu S. Nurellari E. (2023). Silos: an intelligent fault detection scheme for solar insecticidal lamp iot with improved energy efficiency. IEEE Internet Things J. 10, 920–939. doi: 10.1109/JIOT.2022.3209162

[B57] Yang F. Shu L. Yang Y. Han G. Pearson S. Li K. (2021). Optimal deployment of solar insecticidal lamps over constrained locations in mixed-crop farmlands. IEEE Internet Things J. 8, 13095–13114. doi: 10.1109/JIOT.2021.3064043

[B58] Yao H. Shu L. Yang F. Jin Y. Yang Y. (2023). The phototactic rhythm of pests for the solar insecticidal lamp: a review. Front. Plant Sci. 13. doi: 10.3389/fpls.2022.1018711 PMC9893115 36743546

[B59] Zhang X. Bu J. Zhou X. Wang X. (2023). Automatic pest identification system in the greenhouse based on deep learning and machine vision. Front. Plant Sci. 14. doi: 10.3389/fpls.2023.1255719 PMC10568774 37841606

[B60] Zhang X. D. Xing L. F. (2014). The study on intelligent insecticidal lamp with led. Appl. Mechanics Materials 571-572, 985–989. doi: 10.4028/www.scientific.net/AMM.571-572.985

[B61] Zhang H. Zheng L. Huang Z. Song W. (2021). Study on the application of ozone water in greenhouse soil disinfection. J. China Agric. Univ. 26, 189–199. doi: 10.11841/j.issn.1007-4333.2021.11.19

[B62] Zheng Z. Zhang C. (2022). Electronic noses based on metal oxide semiconductor sensors for detecting crop diseases and insect pests. Comput. Electron. Agric. 197, 106988. doi: 10.1016/j.compag.2022.106988

[B63] Zhou J. Long X. Luo H. (2018). Spectrum optimization of light-emitting diode insecticide lamp based on partial discharge evaluation. Measurement 124, 72–80. doi: 10.1016/j.measurement.2018.03.073

